# Evolving Treatment Paradigms in Metastatic Hormone-Sensitive Prostate Cancer: Expert Narrative Review

**DOI:** 10.3390/curroncol32080437

**Published:** 2025-08-05

**Authors:** Vineet Talwar, Kaushal Kalra, Akhil Kapoor, P. S. Dattatreya, Amit Joshi, Krishna Chaitanya, M. V. Chandrakanth, Atul Batra, Krishna Prasad, Nikhil Haridas, Nilesh Lokeshwar

**Affiliations:** 1 Department of Medical Oncology, Rajiv Gandhi Cancer Institute and Research Centre (RGCIRC), New Delhi 110085, India; 2 Department of Medical Oncology, VMMC-Safdarjung Hospital, New Delhi 110029, India; kaushalkalra@yahoo.com; 3 Mahamana Pandit Madanmohan Malaviya Cancer Centre and Homi Bhabha Cancer Hospital, Tata Memorial Centre, Homi Bhabha National Institute, Varanasi 221005, India; akhil@mpmmcc.tmc.gov.in; 4 Medical Oncology Services Renova Hospitals, Hyderabad 500034, India; dnbhead@renovahospitals.com; 5 Department of Medical Oncology, ACTREC, Tata Memorial Centre, HBNI, Mumbai 410210, India; dramitjoshi74@gmail.com; 6 Soumya Multispeciality Hospital, Hyderabad 500015, India; dirmnjiorcc@yahoo.com; 7 Department of Medical Oncology, Narayana Health, Kolkata 700016, India; drmvch@gmail.com; 8 Medical Oncology, Dr. B.R.A. Institute Rotary Cancer Hospital, All India Institute of Medical Sciences (AIIMS), New Delhi 110029, India; batraatul85@aiims.edu; 9 Mangalore Institute of Oncology, Mangalore 575002, India; drkrishnaprasad@hotmail.com; 10 Amrita Institute of Medical Sciences, Amrita Vishwavidyapeedam, Kochi Health Campus, Kochi 682041, India; nikhilkh@aims.amrita.edu; 11 MGM Hospital, Vashi 400703, India; nileshlok@yahoo.com

**Keywords:** metastatic hormone-sensitive prostate cancer, androgen deprivation therapy, triplet therapy, darolutamide, ARSI, treatment sequencing, real-world evidence

## Abstract

The treatment landscape of metastatic hormone-sensitive prostate cancer (mHSPC) has transformed significantly with the advent of triplet therapy involving androgen deprivation therapy (ADT), docetaxel, and androgen receptor signalling inhibitors (ARSIs). While clinical guidelines increasingly support early intensification, real-world practice remains challenged by patient heterogeneity, evolving evidence, and limited consensus on treatment sequencing. This narrative review integrates evidence from landmark trials, clinical guidelines, and expert insights from oncologists managing mHSPC in India. Findings affirm that triplet therapy, particularly with darolutamide, improves survival in high-volume disease and underscores the need for personalized treatment based on disease burden, comorbidities, and genomic profiles. The review also highlights gaps in real-world data, sequencing strategies, and biomarker-driven therapy, reinforcing the need for precision medicine and locally relevant evidence to guide treatment. Ultimately, optimizing mHSPC management requires harmonizing guideline-based approaches with individualized, real-world decision making to improve patient outcomes.

## Simple Summary

Prostate cancer that has spread beyond the prostate but still responds to hormone treatment is known as metastatic hormone-sensitive prostate cancer. Recent advances show that using a combination of hormone therapy, chemotherapy, and newer hormone-blocking drugs can help patients live longer, especially those with more aggressive disease. One such drug, darolutamide, when added to this combination, has shown significant survival benefits in patients with high-volume disease. This review brings together the latest research, treatment guidelines, and expert opinions from Indian doctors. It highlights the importance of tailoring treatment to each patient based on their overall health, cancer burden, and genetic factors. More locally relevant research is needed to help guide doctors in choosing the most effective care for each patient.

## 1. Introduction

Prostate cancer remains the most common type of cancer in males and is recognized as the second leading cause of increased mortality rate among men. According to estimates from the Global Cancer Observatory (GLOBOCAN), there were 19.3 million new cancer cases worldwide in 2020. India ranked third in incidence, following China and the United States [1]. GLOBOCAN indicated that cancer cases in India will rise to 2.08 million by 2040, reflecting a 57.5% increase from 2020 [2]. A 2010 study by the National Cancer Institute estimated that over 11.85 billion dollars were spent in the United States to manage the prevalence of prostate cancer fully [3]. The rate of expenditure is further on the rise with the delayed diagnosis, raising the need for appropriate awareness focused on disease, screening, diagnosis, and treatment. Most of the prostate cancer cases are diagnosed at a localized, hormone-sensitive stage, but still carry a high risk of complications if not appropriately managed. mHSPC presents additional challenges due to complex disease progression, requiring more intensive and individualized treatment strategies [3].

According to the Lancet commission, the late diagnosis of prostate cancer is a significant global issue, particularly in low- and middle-income countries (LMICs), where it remains the norm. To reduce the burden of advanced disease, there is an urgent need to establish structured systems for earlier detection in these regions. Conducting screening trials in LMICs is essential to identify effective strategies for improving early diagnosis and optimizing patient outcomes [4]. In India, 85% of prostate cancer cases are diagnosed in advanced stages (III and IV), whereas in the United States, only 15% of cases are identified in these late stages [5]. Sociodemographic, economic, and healthcare accessibility are the leading contributors. Lack of awareness remains one of the most significant barriers to timely screening, often resulting in delayed diagnosis. An association of the condition with sociodemographic, economic, and geographical factors may further delay this [6]. However, the therapeutic landscape of mHSPC has seen a sea change in the last decade by adding new therapies to improve disease outcomes, both in survival and quality of life.

This paper focuses on the critical review and update on current treatment modalities, clinical guidelines, and emerging therapies, along with expert viewpoints on future directions for mHSPC management. The significance of this review lies in its holistic approach, integrating clinical guidelines, real-world evidence, and expert opinions to optimize treatment strategies. The aim of this review is to bridge the gap between established guidelines and the complexities of real-world practice to provide valuable insights for the optimal management of mHSPC.

## 2. Disease Area: Metastatic Hormone-Sensitive Prostate Cancer (mHSPC)

Globally, over 1.4 million new cases of prostate cancer are diagnosed annually, primarily affecting men between 45 and 74 years of age [7]. mHSPC is characterized by the presence of metastatic lesions in patients whose cancer remains responsive to androgen deprivation therapy (ADT) [3].

The pathophysiology of mHSPC is primarily driven by androgen signalling, which plays a crucial role in prostate cancer cell growth and survival. Androgens, such as testosterone, bind to androgen receptors (ARs) in prostate cells, triggering gene transcription that promotes proliferation and inhibits apoptosis [8].

Metastatic spread significantly affects prognosis and quality of life. Studies have shown that prostate cancer frequently metastasizes to the musculoskeletal system, particularly the vertebral bodies, leading to complications such as impaired mobility, gait disturbances, and increased dependency on daily activities. Additionally, bone metastases often result in severe pain, fatigue, and urinary dysfunction, further impacting patient comfort. The psychological burden is also substantial, with increased rates of anxiety, depression, and a sense of loss of control following a metastatic cancer diagnosis. These physical and emotional challenges contribute to social isolation and diminished overall quality of life [9].

Delayed diagnosis is often associated with poorer outcomes and an increased symptom burden due to widespread metastases. Thus, early detection remains critical since low-volume metastatic disease has a better prognosis compared to high-volume disease [1,10].

## 3. Current Treatment Landscape and Guideline Recommendations

### 3.1. The Overview of Treatment Modalities

The current treatment landscape for mHSPC is characterized by a multimodal approach that includes hormone therapy, chemotherapy, and emerging targeted therapies. Recently, combination therapies involving ADT, androgen receptor signalling inhibitors (ARSIs), and chemotherapy have improved overall survival in hormone-sensitive prostate cancer.

#### 3.1.1. Hormone Therapy

Hormone therapy has been mostly utilized for the treatment of mHSPC. Within hormone therapy, ADT has been the standard treatment for metastatic prostate cancer for many years, and is the cornerstone of mHSPC treatment due to its ability to lower elevated testosterone levels leading to disease suppression [11]. Although ADT can be initiated as part of treatment, successful remission is achieved only through surgical castration or medical therapies such as luteinizing hormone-releasing hormone (LHRH) agonists and antagonists [12]. To summarize, hormone therapy is not curative, and prolonged use can lead to increased resistance, necessitating a transition to alternative treatment modalities.

#### 3.1.2. Chemotherapy

Chemotherapy serves as a secondary treatment modality, aiding in the remission of cancer cells. Docetaxel—the most used—disrupts cell division by stabilizing microtubules. This stabilization interferes with mitotic processes, ultimately inducing apoptosis in rapidly dividing cancer cells [13]. Although docetaxel is administered in combination with ADT routinely, clinical studies have shown the need for alternative/additional treatment modalities [14,15].

#### 3.1.3. Emerging Targeted Therapies—Novel Androgen Receptor Pathway Inhibitors (ARPIs)

**Figure 1:**
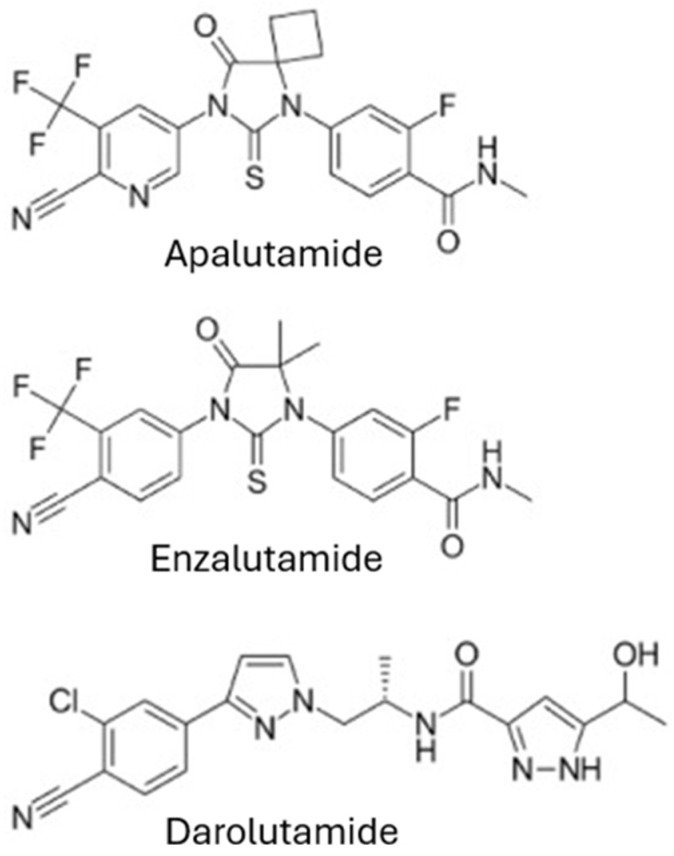
Structures of the ARPIs available on the Indian market.

Emerging therapies are changing the landscape of treatments for mHSPC, providing targeted strategies for managing patients. ARPIs (steroidal and non-steroidal) have been researched since the 1960s. The clinical utility of steroidal ARPIs, such as abiraterone, a P450 c17 (CYP17) enzyme inhibitor, is limited by its adverse effects of cardiac and hepatic toxicity and hypertension. In contrast, non-steroidal ARPIs, including darolutamide, apalutamide, and enzalutamide, are more selective as pure androgen receptor blockers. Unlike abiraterone, their chemical structure is distinct from testosterone and progesterone; they are not associated with progesterone-related adverse effects, making them a safer therapeutic option. ARPIs act by competitively binding to the AR, blocking androgen-mediated signalling. This prevents AR nuclear translocation, co-activator transport, and DNA binding [16]. Figure 1 shows these compounds.

**Figure 2:**
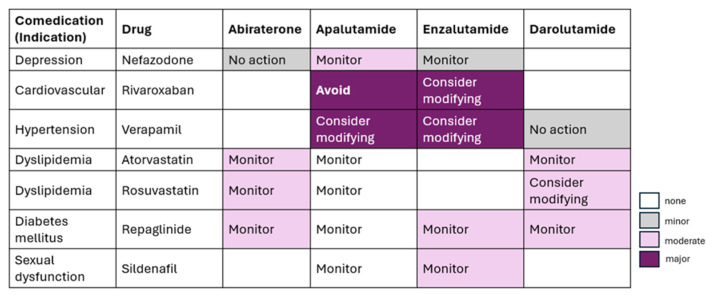
Potential drug–drug interactions between ARPIs.

Darolutamide is a potent non-steroidal ARPI with strong binding and activity against resistant androgen receptor mutations. It has low blood–brain barrier penetration, reducing the risk of CNS-related side effects like seizures. Darolutamide’s safety profile, including the absence of reported seizures in trials, makes it a promising option for advanced prostate cancer, particularly for elderly patients who may be more susceptible to adverse effects from other treatments [11], as shown in Figure 2. Table 1 reflects the review of the latest guidelines.

### 3.2. Effectiveness, Safety, and Patient Outcomes

Therapeutic regimens with traditional hormonal treatment such as ADT with novel targeted agents represent a paradigm shift in the management of mHSPC. By concurrently targeting multiple oncogenic pathways, including the androgen receptor axis and complementary signalling cascades, these combination therapies have demonstrated improved outcomes in phase III clinical trials [11,20]. The synergistic mechanism of action addresses both the hormonal dependence of prostate cancer and emerging resistance pathways, thereby optimizing disease control in mHSPC. While combination therapies offer enhanced clinical benefits, they require careful consideration, as they may result in greater toxicity and a higher incidence of adverse events than monotherapy. To summarize, the benefits must be balanced against the potential risks.

Combination therapies involving ADT with docetaxel or ARPI have demonstrated the longest duration of disease control and improved overall survival compared to other treatment strategies [8]. However, the safety profiles of these regimens differ. ADT combined with docetaxel is associated with a higher incidence of adverse events, including neutropenia, fatigue, and an increased risk of infection. In contrast, ARPIs are generally better tolerated but may lead to hypertension, fatigue, and an increased risk of falls, particularly in elderly patients [20]. Despite such challenges, integrating these therapies has significantly enhanced quality-adjusted life years in patients with mHSPC. Studies continue to optimize these regimens for maximum efficacy with minimal adverse effects to enhance survival and quality of life, mainly optimization of triple therapy.

## 4. Combination Therapy

As discussed before, the treatment landscape for mHSPC is expanding, with various therapeutic combinations now available, including ADT, docetaxel, and next-generation antiandrogens. For decades, ADT monotherapy had been the gold standard for mHSPC treatment. However, recent studies over the past seven years have demonstrated a significant benefit of survival with combination therapies, such as ADT plus docetaxel or ARPIs, extending overall survival by at least 18 months.

Additionally, multiple ongoing phase III trials are evaluating various triple-therapy approaches, incorporating AR-targeting, mitotic inhibitors, immunotherapy, PIk3ca/AKT pathway inhibitors, and poly-ADP ribose polymerase (PARP)/AKT inhibitors. While intensifying treatment with chemotherapy or novel hormonal agents can enhance oncologic outcomes, it may also introduce additional toxicities and financial burdens. As a result, selecting the most appropriate combination therapy also requires the careful consideration of individual patient characteristics and preferences.

### 4.1. Doublet vs. Triplet Therapy

A network meta-analysis and systematic review showed that triplet therapies, specifically darolutamide + docetaxel + ADT and abiraterone + docetaxel + ADT, were ranked highest for overall survival in mHSPC, with hazard ratios of 0.54 and 0.60, respectively. In contrast, doublet therapies like ARPI + ADT and docetaxel + ADT were less effective. In the overall cohort of mHSPC patients, triplet therapy ranked highest (and second highest) in the network meta-analysis for overall survival, with the highest P scores observed for darolutamide + docetaxel + ADT (0.93) and abiraterone + docetaxel + ADT (0.73) [21].

Another study confirmed these findings by highlighting that ADT alone and doublet therapies (docetaxel + ADT) are no longer valid treatment options for mHSPC. For low-volume mHSPC, the benefit of adding a third drug was minimal compared to androgen receptor-axis targeted therapy (ARAT) + ADT. However, in high-volume cases, darolutamide + docetaxel + ADT demonstrated superior survival benefits (HR 0.76) compared to ARAT + ADT, underscoring the importance of triplet therapy in improving patient outcomes [22].

An extensive network meta-analysis of eleven randomized controlled trials involving 11,546 patients revealed that while the triplet therapy (ADT, ARAT, and docetaxel) was ranked as the most effective treatment strategy, it did not show a statistically significant overall survival (OS) improvement over ADT plus ARAT alone (hazard ratio [HR], 0.89; 95% confidence interval [CI], 0.68–1.16). In contrast, ADT plus docetaxel and ADT alone were associated with a higher risk of death [8]. Table 2 presents a summary of various combined therapies and their effectiveness based on real-world evidence.

### 4.2. Patient Profiles

In patients with high-volume and high-risk/low-risk metastatic hormone-sensitive prostate cancer, treatment intensification with darolutamide, ADT, and docetaxel increased OS, consistent with the overall population; the results in the low-volume subgroup were suggestive of survival benefit. Darolutamide should be used with precaution in comorbidities requiring drugs like itraconazole, rifampicin, midazolam, dabigatran, and rosuvastatin due to potential drug–drug interactions [25]. Table 3 summarizes the effect of darolutamide in different patient profiles based on volume and risk of cancer.

## 5. Factors Predicting the Benefit of Intensification of Therapy in mHSPC

The management of mHSPC remains complex, as the optimal level of treatment intensification is not always clear. However, insights from clinical trials and oncologist experiences have helped identify key factors that guide therapy escalation. Disease characteristics such as metastatic volume, location, and aggressiveness, along with patient profiles, play a crucial role in determining the benefit of intensification of therapy [26], as shown in Table 4.

### 5.1. Disease Characteristics and Prior Treatment Response

Disease characteristics play a key role in determining the appropriate treatment approach, including the need for therapy intensification. Another study demonstrated that patients initiated on ADT who responded well had improved survival outcomes. This suggests that an initial response to ADT reflects hormone sensitivity, which can be further leveraged through early combination therapy (e.g., ADT with ARPIs or chemotherapy) to optimize long-term outcomes [9]. Patients with poor initial responses may harbour more resistant diseases that demand alternative strategies. Other genetic markers may also be strong predictors, including changes in the androgen receptor gene or DNA repair genes such as Breast Cancer gene (BRCA) 1/2 [30]. These markers indicate which patients are more likely to respond to intensified therapies, as they can influence the tumour’s biology and susceptibility to different treatments.

### 5.2. Impact of Genetic Markers and Personalized Medicine

Recent advancements in oncology have recognized the critical role of genetic profiling as a predictor of treatment outcomes in the management of mHSPC. Specific genetic alterations, such as mutations in DNA repair genes (BRCA1 (Breast Cancer 1), BRCA2 (Breast Cancer 2), ATM (Ataxia-Telangiectasia Mutated)), predict treatment outcomes and guide therapy-related decisions. These mutations, when combined with ADT and ARPIs, increase sensitivity to PARP inhibitors, thereby improving patient responses [31]. The early detection of genetic alterations enables individualized treatment with PARP inhibitors, enhancing therapeutic response and prolonging progression-free survival. These advancements mark significant progress toward personalized medicine in mHSPC, shifting from a one-size-fits-all approach to therapies tailored to the tumour’s molecular profile. Furthermore, the application of such genetic markers in treatment planning is not limited to PARP inhibitors but extends to other therapeutic options. For example, alterations in the AR gene may predict resistance to ARPIs, suggesting alternative therapeutic strategies for these patients. This approach is poorly utilized in real-world clinical practice. Epigenetic factors, such as DNA methylation, histone modifications, and chromatin remodelling, also affect prostate cancer progression and response to treatment by altering gene expression and promoting therapy resistance [32]. Further research is needed to validate these genetic and epigenetic markers and investigate the most effective way of integrating them into treatment protocols [8].

## 6. Future Directions

### 6.1. Gaps in Current Treatment Options

Even though there have been substantial recent therapeutic advances in mHSPC, currently available therapeutic options are not adequate in covering all the gaps. A notable gap is the insufficient efficacy of regimens in high-volume or aggressive disease settings. Although combinations, such as ADT with docetaxel or ARPI, do improve survival rates, benefits from these options are not universal. For instance, certain patients display primary resistance to these therapies, characterized by early failures of the treatment and, consequently, disease progression [33]. The variability in response underlines a crucial unmet need for more effective treatment options to consistently and reliably manage the different populations of patients. Moreover, the heterogeneity of prostate cancer is influenced by genetic mutations, tumour microenvironment, and molecular traits, which makes the elaboration of a universal strategy complicated and leaves a large proportion of patients without the proper therapeutic interventions [15]. Emerging evidence suggests that among patients classified as high-volume, those with visceral pulmonary metastases may have a more favourable prognosis compared to those with liver metastases or multiple bone metastases. Similarly, factors such as very high PSA at diagnosis, Gleason scores of 9–10 indicating poorly differentiated tumours, or extensive lymph node involvement may influence treatment decisions toward triplet therapy [34].

Other unmet needs are represented by the development of resistance to ARPI and chemotherapy, which are still some of the biggest challenges in managing mHSPC. Other proposed resistance mechanisms include alterations in androgen receptors, the activation of alternative survival pathways, and the epithelial-to-mesenchymal transition, contributing to the low durability of current therapies [15]. As patients progress to castration-resistant prostate cancer, the choices of options become decidedly limited, with generally poorer prognosis and fewer effective interventions available. The current therapeutic landscape also lacks personalized approaches for individual patients’ unique molecular and genetic profiles. Precision medicine, which tailors treatment to specific biomarkers or genetic alterations, is promising; however, it has not yet been fully integrated into routine clinical practice for mHSPC [33]. This therapeutic gap still calls for continued research in novel therapeutic targets and personalized treatment strategies that can hopefully strengthen outcomes for all patients with mHSPC.

### 6.2. Sequencing of ARPI Therapeutic Options

The sequencing of ARPI therapeutic options in the treatment of mHSPC remains complex and dynamic. Current clinical guidelines suggest several combinations and sequences of therapies; however, a consensus has not been achieved on the optimum approach. For example, the timing and sequence of ARPI therapy about chemotherapy can make all the difference in outcomes [35]. Some postulate that initiating ARPI before chemotherapy may be more beneficial for overall survival, especially in low-disease-burden patients. Others have countered that this approach may not be as effective as starting with chemotherapy and then proceeding to ARPI in this highly selected population when aggressive diseases, by necessity, must receive cytotoxic intervention upfront [36]. This variability in treatment responses, therefore, underlines the importance of a more personalized approach to sequencing, which would be tailored according to the distinctive clinical characteristics of each patient.

Furthermore, the complexity of the ARPI sequencing strategy is added due to many factors involving the comorbid situation of the patient, treatment history, and tumour biology. Patients with a history of cardiovascular problems are less apt for certain therapies of the ARPI; this means that another sequence will be necessary to avoid side effects. Also, the knowledge of the tumour’s molecular profile, such as unique genetic mutations, can guide the efficacy of specific ARPI sequences, which necessitates a personalized line of treatment [8]. Although substantial evidence exists, definitive data on the optimal sequencing of ARPI therapies remain limited. Clinical decisions may vary, even within standard protocols, showing the complexity of treatment selection. It is therefore crucial to proceed to further research on the most effective ways of sequencing to optimize treatment effectiveness while minimizing resistance and improving overall patient outcomes in mHSPC [36].

### 6.3. Clinician Perspectives on Therapy Prioritization

Clinicians’ views on prioritizing therapy for mHSPC shape the treatment landscape. Based on clinician preference, clinical experience, patient demographics, and institutional resource availability, such views remain divergent. Many clinicians taking care of a high number of patients with high-volume or aggressive mHSPC are biassed toward intensive combination therapies early during the disease, particularly toward the intensification of ADT with chemotherapy or ARPI. This is usually based on the belief that the earlier the aggressive treatment of the tumour, the better the overall survival [23]. Clinicians adopting such a strategy often base any modification on studies which have shown the superiority of the early initiation of therapy, especially in high-burden patients with a given disease, because delayed aggressive therapy may allow for earlier progression [33]. However, this approach has its risks in the form of increased toxicity or side effects, which could adversely affect the quality of life of the patient.

On the other hand, some clinicians favour a more conservative approach to the prioritization of therapy, especially in patients with lower disease burden or with major comorbidities. As can be seen from this viewpoint, overly aggressive treatment could cause overtreatment, thereby leading to irrelevant side effects and a reduction in the patient’s quality of life without a concordant gain in changes in survival [12]. Weighing the potential benefits of early intensive therapy against the risk of adverse effects, specifically in more age- or health-vulnerable patients, balances the risks of adverse effects of early intensive treatment. A conservative approach is increasingly taken regarding patient preferences and quality of life, emphasizing shared decision making about treatment. The divergence in opinion between clinicians indicates a continued debate in the medical community over how to prioritize strategies for therapies in mHSPC and, hence, an indication of a pressing priority with a critical need for expanded clinical trials and guidelines, which could give more detail in treating these patients under the different patient subgroups [33].

## 7. Expert Insights

A review was conducted by collecting inputs through an online survey form completed by 10 experts specializing in oncology. These experts were chosen based on their extensive clinical experience and research contributions to prostate cancer management, ensuring diverse perspectives on current practices and emerging trends. The survey aimed to gather expert views on several critical aspects of prostate cancer care, including screening strategies, diagnostic protocols, treatment options, and supportive care practices.

### 7.1. Prostate Cancer Screening and Early Detections

Experts emphasized the urgent need to address the rising incidence of prostate cancer in India, particularly among men aged 50 and above. A majority supported Prostate Specific Antigen (PSA) screening for men over 50 years, and 70% recommended earlier screening (starting at 45) for those with a family history or BRCA mutations. There was also strong agreement (90%) that low-risk patients with elevated PSA but no other risk factors should have avoided unnecessary biopsies. Insights from the National Cancer Registry Programme also agree that prostate cancer incidence showed a rise after the age of 50, with a notable acceleration after age 64 [37].

### 7.2. Treatment Strategies for mHSPC Patients

For high-volume mHSPC, 80% of experts agreed that triplet therapy (ADT, docetaxel, and darolutamide/enzalutamide) should have been the first-line treatment. However, triplet therapy for metachronous low-volume mHSPC was met with reluctance, favouring doublet therapy instead. There was unanimous agreement (90%) on intensifying treatment for high-volume mHSPC, irrespective of the initial response. Treatment was recommended to be adapted for frail or elderly patients based on comorbidities, and cost-effectiveness was considered, especially in LMICs like India.

Reports from studies indicate that while treatment pursued with a combination of ADT and ARPI or chemotherapy, in the beginning, brings about significantly better overall survival, particularly for patients having high-volume disease, this approach can hit the primary tumour better and, at the same time, may delay the growth of metastatic lesions, extending the time to resistance [33]. Early intensification is particularly emphasized in cases where the disease burden is high, or patient health status permits more aggressive treatment. The rationale of this approach is to maximize the therapeutic effect at a stage when the cancer is most sensitive concerning treatment, with the potential for longer-term disease control. Experts also caution that such an early intensification of treatment must be very individualized, considering even the comorbid conditions and genetic markers of the patient, to avoid overtreatment with any potential unnecessary toxicity. While the survival benefits are undisputed in subgroups of patients with high-volume disease, the approach may not apply universally. For example, those patients with low-volume disease or serious comorbidities would poorly bear the side effects of intensified treatment [35].

### 7.3. Genetic Profiling and Molecular Targeting

Experts advocated for sequencing based on molecular profiling, with almost all of them supporting the use of PARP inhibitors for patients with DNA repair mutations. Additionally, many experts endorsed routine Homologous Recombinant Repair gene testing at diagnosis to guide treatment decisions. A study highlighted the urgent need to address Homologous Recombinant Repair (HRR) gene alterations early in the treatment course of mHSPC. Patients with pathogenic HRR alterations, particularly in BRCA2 and CDK12, experienced a significantly shorter time to progression to castration-resistant prostate cancer mCRPC compared to those without such alterations (12.7 vs. 16.1 months, HR 1.95, *p* = 0.02). These findings suggested that early identification and intervention targeting HRR deficiencies, such as with PARP inhibitors, could improve outcomes. Prospective studies were deemed essential to validate these approaches and optimize treatment strategies for this high-risk population [24].

### 7.4. Real-World Evidence and Localized Data

There is a scarcity of real-world evidence in India, indicating a pressing need for further research to inform and optimize treatment guidelines for mHSPC patients in the region. There was unanimous agreement among the experts on the need for and importance of real-world evidence, particularly from Indian populations, in shaping treatment guidelines for mHSPC.

### 7.5. Supportive Care and Patient-Cantered Approaches

All experts agreed that supportive care, including pain management and psychosocial support, should have been integrated into routine mHSPC treatment. Comprehensive patient counselling was considered essential for improving quality of life, particularly when addressing side effects and treatment intensification. A review emphasized the need for additional, patient-centred, culturally relevant supportive care interventions. Collaboration between nurse scientists and clinicians is crucial to develop interventions that address diverse needs, guided by robust frameworks and validated measurement tools [38].

### 7.6. Emerging and Advanced Therapies

Combination therapies were the most preferred, while immunotherapy was the least favoured among the experts. Radioligand therapy (e.g., Lutetium-177 PSMA) was considered an important option for post-triplet therapy progression, alongside ongoing treatments. Radioligand therapy, such as Lutetium-177 PSMA-617, has shown promise in treating advanced prostate cancer, particularly after progression following conventional therapies. Studies indicate that this therapy can improve survival and quality of life, making it a valuable option for patients with limited treatment choices [39,40].

### 7.7. Biomarkers and Advanced Diagnostics

Experts agreed that current biomarkers like PSA, androgen receptor expression, and BRCA1/BRCA2 mutation could guide therapy selection, enhancing treatment outcomes.

### 7.8. Oligometastatic Prostate Cancer

For oligometastatic prostate cancer, experts agreed that radiotherapy should be included alongside systemic treatments for optimal management. The improvement in outcomes with the treatment of primary and metastatic sites is likely due to reduced tumour burden, with radiotherapy (RT) enhancing ADT through mechanisms like inducing double-strand DNA breaks that lead to apoptosis, as also supported by European Association of Urology and the European Organization for Research and Treatment of Cancer [41].

## 8. Conclusions

In conclusion, novel medicines and disease biology have improved mHSPC care. This study shows that early treatment intensification improves outcomes for mHSPC patients. Early treatment with ARPI and chemotherapy have improved survival, especially for patients with high-volume or aggressive disease [33]. Patient responses vary, highlighting the necessity for tailored therapy based on tumour biology, comorbidities, and health conditions [8]. With an increasing tendency toward aggressive early treatments, clinician perceptions of therapeutic priorities also influence treatment choices [23]. Despite these advances, ARPI sequencing is still debated, and additional clinical studies are needed to standardize therapy protocols [36]. Personalized medicine for mHSPC therapy is still developing, making it difficult to adjust treatments to specific genetic profiles [15]. Early intensification shows promise, but more research and education are needed to enhance treatment procedures and patient outcomes.

Future directions in managing mHSPC will likely be pointing out the gaps in the therapeutic options available, where one can provide a better sequencing of ARPI therapies. Novel therapy approaches to be developed through research will help deliver therapeutic strategies overcoming the stiffness of many currently available treatments resistant to aggressive diseases and give more sustainable responses to the patients [8]. Personalized medicine has a big role in developing genomic profiling, leading to multiple details of treatment plans for more effective results than ever before. Also, in the future, clinical trials should strive to set clearer parameters on the ideal sequencing for ARPI and other therapies to minimize outcome variance [36]. Such combination therapies that involve a novel agent administered in combination with available treatments could further the effectiveness of the treatment and reduce resistance. In addition, building on the educational initiatives in this area will provide clinicians with the best means to translate the latest study findings to clinical practice moving forward [15]. Ultimately, the goal is to achieve more personalized, effective, patient-centred care for mHSPC patients.

## Figures and Tables

**Table 1:** Review of the latest guidelines.

Guideline	Recommendation
National Comprehensive Cancer Network (NCCN), 2024 [17]	ADT + Abiraterone or Darolutamide (Category 1) for high-volume synchronous, high-volume metachronous, or low-volume synchronous metastases
American Urological Association (AUA), 2023 [18]	ADT + ARPIs (Abiraterone + Prednisone, Apalutamide, Enzalutamide) or Docetaxel (Grade A); Selected de novo mHSPC: ADT + Docetaxel + Abiraterone or Darolutamide
Urology Society of India, 2023 [19]	ADT + Docetaxel or Novel ARPIs (Darolutamide, Enzalutamide, Apalutamide) for mHSPC

**Table 2:** Summary of various combined therapies and their effectiveness based on real-world evidence.

Combined Therapy	Effectiveness
ADT + Docetaxel	Improves overall survival, particularly in patients with high-volume disease; delays progression to CRPC [21,23]
ADT + Abiraterone	Significantly improves overall survival and radiographic progression-free survival; beneficial in high-risk patients [9,11]
ADT + Enzalutamide	Prolongs progression-free and overall survival; reduces the risk of progression to CRPC [7,24]
ADT + Apalutamide	Extends survival and the time to metastasis and benefits both low- and high-risk disease groups [9,24]
ADT + Darolutamide	Improves progression-free survival and overall survival with a favourable safety profile; delays the onset of CRPC [8,23]
ADT + Docetaxel + ARPI (e.g., Darolutamide)	Offers a triple-therapy approach that maximizes survival benefits, particularly effective in high-volume and high-risk patients [9,21]

**Table 3:** Effect of darolutamide in different patient profiles based on volume and risk of cancer according to Hussain et al., 2023 [23].

Parameter	High Volume	Low Volume	High Risk	Low Risk
Overall Survival (OS)	31% risk reduction (HR 0.69)	32% risk reduction (HR 0.68)	29% risk reduction (HR 0.71)	38% risk reduction (HR 0.62)
Time to Castration-Resistant Prostate Cancer	Improved	Improved	Improved	Improved
Time to Initiation of Subsequent Therapy	Improved	Improved	Improved	Improved
Pain Progression and Skeletal Events	Improved (HR < 1)	Improved (HR < 1)	Improved (HR < 1)	Improved (HR < 1)

**Table 4:** Key predictive factors for intensified therapy in mHSPC.

Predictive Factor	Definition/Criterion	Impact on Treatment Decision
Disease Volume [27]	High-volume: Visceral metastases and/or ≥4 bone metastases	Triplet therapy (ADT + Docetaxel + ARSI) recommended
Disease Risk [28]	High-risk: Gleason ≥8, ≥3 bone lesions, visceral metastases	Higher likelihood of benefiting from intensified therapy
De Novo vs. Recurrent Disease [26]	De Novo: metastases at diagnosis Recurrent: after local therapy	De Novo: worse prognosis → needs aggressive treatment (Triplet Therapy) Recurrent: may benefit from doublet (ADT + ARSI)
Triplet Therapy (ADT + Docetaxel + ARSI) [23,29]	Proven survival benefit in high-volume, high-risk mHSPC	Standard for chemotherapy-fit patients. Darolutamide shown to improve OS when added to Docetaxel + ADT
Doublet Therapy (ADT + ARSI) [26]	ADT + Enzalutamide, Apalutamide, Abiraterone, or Darolutamide	Preferred in low-volume or recurrent disease, where Docetaxel benefit is unclear
Radiotherapy for Low-Volume Disease [26]	Prostate-directed radiation in low-volume, de novo mHSPC	Improves survival, especially when combined with ADT + ARSI

Abbreviations: mHSPC: metastatic hormone-sensitive prostate cancer; ADT: androgen deprivation therapy; ARSI: androgen receptor signalling inhibitor; OS: overall survival; ARPI: androgen receptor pathway inhibitor.

## Abbreviations

The following abbreviations are used in this manuscript:

mHSPC	Metastatic Hormone-Sensitive Prostate Cancer
ADT	Androgen Deprivation Therapy
ARSI	Androgen Receptor Signalling Inhibitor
OS	Overall Survival
ARPI	Androgen Receptor Pathway Inhibitor
